# Supplementary material for: Multi‐dataset identification of innovative feature genes and molecular mechanisms in keratoconus
Source: J Cell Mol Med. 2024 Sep 19;28(18):e70079. doi: 10.1111/jcmm.70079 (PMC11412914; doi:10.1111/jcmm.70079)
Supplement: Supplementary file 2 — Material S2. [file JCMM-28-e70079-s002.zip › 7.GSEA analysis/5.RNF217-AS1/KEGG_GNRH_SIGNALING_PATHWAY.html]

Details for gene set KEGG\_GNRH\_SIGNALING\_PATHWAY[GSEA]

|  || Dataset | RNF217-AS1.group.cls#h\_versus\_l |
| Phenotype | group.cls#h\_versus\_l |
| Upregulated in class | h |
| GeneSet | KEGG\_GNRH\_SIGNALING\_PATHWAY |
| Enrichment Score (ES) | 0.19223249 |
| Normalized Enrichment Score (NES) | 0.8592007 |
| Nominal p-value | 0.71602434 |
| FDR q-value | 1.0 |
| FWER p-Value | 1.0 |
Table: GSEA Results Summary

  

Fig 1: Enrichment plot: KEGG\_GNRH\_SIGNALING\_PATHWAY      
 Profile of the Running ES Score & Positions of GeneSet Members on the Rank Ordered List

  

| SYMBOL | TITLE | RANK IN GENE LIST | RANK METRIC SCORE | RUNNING ES | CORE ENRICHMENT || 1 | CALML6 | na | 1514 | 0.391 | -0.0076 | Yes |
| 2 | GNRH1 | na | 1533 | 0.389 | 0.0277 | Yes |
| 3 | CGA | na | 2795 | 0.300 | 0.0190 | Yes |
| 4 | ADCY2 | na | 2970 | 0.293 | 0.0410 | Yes |
| 5 | CAMK2A | na | 3603 | 0.267 | 0.0474 | Yes |
| 6 | FSHB | na | 3675 | 0.264 | 0.0697 | Yes |
| 7 | ADCY4 | na | 4384 | 0.238 | 0.0712 | Yes |
| 8 | GNRHR | na | 4497 | 0.234 | 0.0895 | Yes |
| 9 | ADCY5 | na | 4777 | 0.225 | 0.1022 | Yes |
| 10 | PLA2G4E | na | 4967 | 0.218 | 0.1169 | Yes |
| 11 | CAMK2B | na | 5000 | 0.217 | 0.1359 | Yes |
| 12 | CACNA1F | na | 5544 | 0.198 | 0.1385 | Yes |
| 13 | ADCY7 | na | 5579 | 0.197 | 0.1556 | Yes |
| 14 | PLA2G2F | na | 5587 | 0.196 | 0.1735 | Yes |
| 15 | GNAQ | na | 7033 | 0.152 | 0.1458 | Yes |
| 16 | CACNA1S | na | 7170 | 0.148 | 0.1555 | Yes |
| 17 | PLCB3 | na | 7444 | 0.141 | 0.1606 | Yes |
| 18 | PLA2G2C | na | 7467 | 0.141 | 0.1730 | Yes |
| 19 | CACNA1C | na | 7536 | 0.139 | 0.1839 | Yes |
| 20 | PLA2G2D | na | 7735 | 0.134 | 0.1905 | Yes |
| 21 | ELK1 | na | 8380 | 0.119 | 0.1829 | Yes |
| 22 | HBEGF | na | 8507 | 0.116 | 0.1900 | Yes |
| 23 | CACNA1D | na | 8999 | 0.105 | 0.1855 | Yes |
| 24 | MAPK12 | na | 9095 | 0.103 | 0.1922 | Yes |
| 25 | SOS1 | na | 9467 | 0.096 | 0.1904 | No |
| 26 | MAP3K4 | na | 10181 | 0.083 | 0.1774 | No |
| 27 | MMP14 | na | 10390 | 0.080 | 0.1788 | No |
| 28 | PLA2G12B | na | 10412 | 0.079 | 0.1855 | No |
| 29 | ADCY1 | na | 10624 | 0.076 | 0.1864 | No |
| 30 | JMJD7-PLA2G4B | na | 10713 | 0.074 | 0.1907 | No |
| 31 | MAPK13 | na | 10953 | 0.071 | 0.1903 | No |
| 32 | KRAS | na | 11445 | 0.064 | 0.1820 | No |
| 33 | MAPK11 | na | 12121 | 0.054 | 0.1675 | No |
| 34 | ITPR3 | na | 12500 | 0.049 | 0.1611 | No |
| 35 | MAPK9 | na | 12895 | 0.044 | 0.1537 | No |
| 36 | LHB | na | 13286 | 0.039 | 0.1460 | No |
| 37 | MAPK1 | na | 13362 | 0.038 | 0.1474 | No |
| 38 | SRC | na | 13502 | 0.036 | 0.1467 | No |
| 39 | CALM2 | na | 13884 | 0.032 | 0.1386 | No |
| 40 | PTK2B | na | 14053 | 0.030 | 0.1365 | No |
| 41 | MAPK3 | na | 14127 | 0.029 | 0.1371 | No |
| 42 | PLA2G10 | na | 14207 | 0.028 | 0.1375 | No |
| 43 | MAPK14 | na | 14311 | 0.027 | 0.1370 | No |
| 44 | MAP3K2 | na | 14371 | 0.026 | 0.1377 | No |
| 45 | PLD2 | na | 14408 | 0.026 | 0.1390 | No |
| 46 | MAP2K7 | na | 14547 | 0.024 | 0.1372 | No |
| 47 | ATF4 | na | 14884 | 0.020 | 0.1294 | No |
| 48 | PLA2G2E | na | 14946 | 0.020 | 0.1295 | No |
| 49 | CALML5 | na | 15459 | 0.014 | 0.1160 | No |
| 50 | PRKCD | na | 15568 | 0.013 | 0.1141 | No |
| 51 | MAPK10 | na | 15620 | 0.013 | 0.1138 | No |
| 52 | HRAS | na | 15878 | 0.010 | 0.1073 | No |
| 53 | CAMK2G | na | 16021 | 0.008 | 0.1040 | No |
| 54 | MAP2K2 | na | 16236 | 0.006 | 0.0984 | No |
| 55 | MAP2K4 | na | 16303 | 0.006 | 0.0970 | No |
| 56 | GNA11 | na | 16472 | 0.004 | 0.0926 | No |
| 57 | PLA2G6 | na | 16529 | 0.003 | 0.0913 | No |
| 58 | MAPK8 | na | 16681 | 0.002 | 0.0871 | No |
| 59 | MAPK7 | na | 16971 | -0.001 | 0.0788 | No |
| 60 | PLA2G4A | na | 17031 | -0.002 | 0.0773 | No |
| 61 | MAP2K3 | na | 17092 | -0.003 | 0.0758 | No |
| 62 | ADCY9 | na | 17206 | -0.004 | 0.0729 | No |
| 63 | NRAS | na | 17304 | -0.005 | 0.0705 | No |
| 64 | CALML3 | na | 17310 | -0.005 | 0.0708 | No |
| 65 | GNAS | na | 17807 | -0.009 | 0.0573 | No |
| 66 | CDC42 | na | 18054 | -0.011 | 0.0513 | No |
| 67 | CALM3 | na | 18318 | -0.014 | 0.0449 | No |
| 68 | RAF1 | na | 18969 | -0.020 | 0.0280 | No |
| 69 | GNRH2 | na | 19126 | -0.021 | 0.0254 | No |
| 70 | ADCY6 | na | 19355 | -0.024 | 0.0210 | No |
| 71 | SOS2 | na | 19710 | -0.027 | 0.0133 | No |
| 72 | CAMK2D | na | 20347 | -0.033 | -0.0020 | No |
| 73 | PLA2G12A | na | 20757 | -0.037 | -0.0104 | No |
| 74 | PLCB4 | na | 21374 | -0.044 | -0.0241 | No |
| 75 | PLD1 | na | 21493 | -0.045 | -0.0233 | No |
| 76 | ADCY8 | na | 21903 | -0.050 | -0.0306 | No |
| 77 | ITPR2 | na | 21984 | -0.051 | -0.0282 | No |
| 78 | MAP3K3 | na | 22073 | -0.052 | -0.0260 | No |
| 79 | GRB2 | na | 22374 | -0.055 | -0.0296 | No |
| 80 | ADCY3 | na | 22794 | -0.059 | -0.0362 | No |
| 81 | PRKACA | na | 23066 | -0.063 | -0.0383 | No |
| 82 | MAP2K1 | na | 23215 | -0.064 | -0.0367 | No |
| 83 | PRKCA | na | 23805 | -0.072 | -0.0470 | No |
| 84 | EGFR | na | 24191 | -0.078 | -0.0509 | No |
| 85 | MAP3K1 | na | 24706 | -0.086 | -0.0578 | No |
| 86 | ITPR1 | na | 25459 | -0.098 | -0.0705 | No |
| 87 | MMP2 | na | 25762 | -0.103 | -0.0698 | No |
| 88 | PRKACB | na | 26457 | -0.116 | -0.0792 | No |
| 89 | CALM1 | na | 26557 | -0.117 | -0.0712 | No |
| 90 | JUN | na | 27259 | -0.131 | -0.0794 | No |
| 91 | PLA2G1B | na | 29143 | -0.180 | -0.1171 | No |
| 92 | PLA2G5 | na | 30416 | -0.220 | -0.1335 | No |
| 93 | PLCB2 | na | 31149 | -0.248 | -0.1318 | No |
| 94 | PRKX | na | 31362 | -0.257 | -0.1142 | No |
| 95 | MAP2K6 | na | 32259 | -0.307 | -0.1118 | No |
| 96 | PLCB1 | na | 32371 | -0.315 | -0.0860 | No |
| 97 | PLA2G2A | na | 33013 | -0.361 | -0.0712 | No |
| 98 | PRKACG | na | 33174 | -0.374 | -0.0414 | No |
| 99 | PLA2G3 | na | 33574 | -0.415 | -0.0147 | No |
| 100 | PRKCB | na | 34175 | -0.520 | 0.0160 | No |
Table: GSEA details [plain text format]

  

Fig 2: KEGG\_GNRH\_SIGNALING\_PATHWAY      
 Blue-Pink O' Gram in the Space of the Analyzed GeneSet

  

Fig 3: KEGG\_GNRH\_SIGNALING\_PATHWAY: Random ES distribution      
 Gene set null distribution of ES for **KEGG\_GNRH\_SIGNALING\_PATHWAY**
